# Cytoprotective and Antioxidant Effects of an Edible Herb, *Enhydra fluctuans* Lour. (Asteraceae), against Experimentally Induced Lead Acetate Intoxication

**DOI:** 10.1371/journal.pone.0148757

**Published:** 2016-02-09

**Authors:** Tarun K. Dua, Saikat Dewanjee, Ritu Khanra, Swarnalata Joardar, Sujata Barma, Shilpa Das, M. Zia-Ul-Haq, Vincenzo De Feo

**Affiliations:** 1 Advanced Pharmacognosy Research Laboratory, Department of Pharmaceutical Technology, Jadavpur University, Kolkata 700032, India; 2 The Patent Office, Karchi, 75400, Pakistan; 3 Department of Pharmacy, University of Salerno, 84084 Fisciano, Salerno, Italy; Indian Institute of Toxicology Research, INDIA

## Abstract

**Background:**

*Enhydra fluctuans* Lour. (Asteraceae), an edible aquatic herb, is traditionally employed against toxic effects of heavy metals in India. The present study was planned to discover the protective effect of edible extract of *E*. *fluctuans* (AEEF) against Pb toxicity.

**Methods:**

The cytoprotective role of AEEF was determined on murine hepatocytes employing MTT assay and Hoechst staining. The effects on lipid peroxidation, protein carbonylation, endogenous redox systems and the transcription levels of apoptotic proteins were studied after incubating the hepatocytes with AEEF (400 μg/ml) + Pb-acetate (6.8 μM). The defensive role of AEEF (100 mg/kg) against Pb-acetate (5 mg/kg) intoxication was measured in mice by *in vivo* assays. Biochemical, haematological and histological parameters, intracellular Pb burden and redox status were measured.

**Results:**

AEEF exhibited a concentration dependent cytoprotective effect against Pb-induced cytotoxicity *in vitro*. Pb-acetate incubation significantly (p < 0.01) altered the extents of ROS production ↑, protein carbonylation ↑, lipid peroxidation ↑, endogenous antioxidant enzymes ↓ and GSH ↓ *in vitro*. Besides, Pb-acetate significantly (p < 0.01) induced apoptosis in the hepatocytes apparent from the altered expressions of apoptotic proteins viz. Apaf-1 ↑, Bad ↑, Bcl-2 ↓, Cyt C ↑, cleaved caspases↑, Bid ↑ and Fas ↑. However, AEEF (400 μg/ml) could significantly (p < 0.05–0.01) attenuate the Pb-acetate mediated toxic manifestation *in vitro*. In *in vivo* assay, Pb-acetate (5 mg/kg) treated mice exhibited significantly (p < 0.01) high intracellular Pb content. A high Pb-burden within the tissues caused significant (p < 0.05–0.01) patho-physiological alterations viz. ROS production ↑, protein carbonylation↑, lipid peroxidation ↑, DNA fragmentation ↑, ATP formation ↑, mitochondrial co-enzymes Q ↓, endogenous antioxidant enzymes ↓ and GSH ↓ within the selected tissues. The haematological and serum biochemical parameters were significantly (p < 0.05–0.01) different in the Pb-acetate treated mice. Finally, histological assessment imposed significant toxic occurrence within the organs of Pb-intoxicated animals. However, concurrent administration of AEEF (100 mg/kg) could significantly (p < 0.05–0.01) reinstate the Pb-acetate mediated toxicity.

**Conclusion:**

Presence of metal chelators and phyto-antioxidants within AEEF would offer overall protection through promoting Pb clearance coupled with restoring redox balance.

## Introduction

Lead (Pb), a toxic heavy metal, imposes great environmental concern with its severe acute and chronic toxic manifestations in different organs and various systems within the organisms [1]. Pb is known to induce an array of pathological incidences/abnormalities in the laboratory animals and humans [2]. Accelerated employment of Pb-based consumables and industrial outcomes results in the contamination of the air, dust and soils with Pb-compounds potentially responsible for the Pb pollution [3]. The non-biodegradable nature of Pb-compounds ensures the prolonged persistence of Pb in the environment. Despite Pb is a non-redox metal, it imparts pathogenesis via oxidative disturbances through the generation of excessive oxidative radicals coupled with the depletion of the cellular antioxidant defense [2,4]. Pb does not possess a direct pro-oxidant catalytic activity [5]. Therefore, it imparts oxidative challenges by some indirect mechanisms. Earlier reports revealed that, Pb-mediated auto-oxidation of haemoglobin, δ-aminolevulinic acid (ALA) accumulation and auto-oxidation of ALA are responsible for the over-production of ROS [5]. Pb exhibits electron sharing capability to form covalent linkage with -SH groups of the antioxidant enzymes, which are the most potential targets of Pb [6]. Besides, Pb hampers the critical cellular balances of some important trace elements which are essential for the catalytic activities of endogenous antioxidant enzymes. By these ways, Pb causes the imbalance of endogenous redox defense system [7]. Later render the accumulation of excessive ROS within the cells. ROS participate in the direct oxidative damages of the structural and functional macromolecules within the cells and induces apotototic cell death [8]. Despite different metal chelating agents are commercially available, the contraindication principally redistribution and translocation of Pb into another organs largely confined their applications [5]. Among different side effects, nephrotoxicity (CaNa_2_EDTA), hypersensitivity (succimer, BAL), cardio-toxicity (BAL), zinc dieresis (CaNa_2_EDTA), haematotoxicity (D-penicillamine) nausea, fever, breathing trouble etc. have been demonstrated [5]. As discussed in this section, the induction of ROS coupled with disturbances in the cellular redox defense encouraged to exploit the defensive effect of a dietary antioxidant against chemically induced Pb-toxicity. Exogenously supplied anti-oxidants would be proven beneficial to rebalance the impaired pro- and anti-oxidant ratio due to Pb-intoxication.

*Enhydra fluctuans* Lour. (Asteraceae) is an aquatic herb distributed in South Eastern Asia, India, and China. It is commonly consumed as green leafy vegetables and it possesses high nutritive values to human beings. *E*. *fluctuans* has been used in conventional medicine against various diseases namely inflammation, pain, liver diseases, diabetes, intestinal warm, high blood pressure, heavy metal toxicity [9–13] etc. The existing literature revealed presence of phenolic compounds, saponins, β-carotene and ascorbic acid in the aerial part of *E*. *fluctuans* [10,14]. The present study has been executed to evaluate the possible protective role of an edible (aqueous) extract of the aerial part of *E*. *fluctuans* against experimentally induced Pb-toxicity. The protective role of the test extract has been measured employing murine hepatocytes (*in vitro*) and mice (*in vivo*) models. It has been further aimed to find out the possible protective mechanism(s) and to demonstrate the correlation between the observed effects with respect to phyto-constituents present within the *E*. *fluctuans*.

## Materials and Methods

### Chemicals

Bradford reagent, bovine serum albumin, fetal bovine serum (FBS), Collagenase type I, Dulbecco’s modified Eagle’s medium (DMEM), primary antibodies and HRP conjugated secondary antibody were acquired from Sigma-Aldrich Chemical, St. Louis, USA. HPLC grade solvents viz. methanol, formic acid, acetic acid and acetonitrile were obtained from Merck, India. Pb-acetate, ethylenediaminetetraacetic acid (EDTA), ammonium sulphate, sodium azide, potassium dihydrogen phosphate (KH_2_PO_4_), sodium pyrophosphate, glacial acetic acid, 1-chloro-2,4-dinitrobenzene, 2,4-dinitro-phenyl-hydrazine, 5,5-di-thio-bi(2-nitrobenzoic acid), nitro blue tetrazolium (NBT), N-ethylmaleimide, reduced nicotinamide adenine dinucleotidereduced disodium salt, phenazinemethosulphate, trichloro acetic acid thiobarbituric acid, 5-thio-2-nitrobenzoic acid andreduced glutathione, were obtained from Sisco Research Laboratory, India. Kits for measurement of total cholesterol, creatinine kinase (CK), lactate dehydrogenase (LDH) andtriglycerides were bought from Span Diagnostic Ltd., India.

### Preparation of extract

The dried and pulverized aerial parts of *E*. *fluctuans* were drenched with distilled water containing chloroform (1%) for 2 days at 30 ± 5°C with continuous stirring [10]. The extract obtained was sieved to remove cellular debris and the filtrate was lyophilized to get the dried crude extract of AEEF (~12.5%, w/w) [10]. AEEF was liquefied in distilled water containing tween 80 (1%) before *in vivo* experiment. For *in vitro* tests, the AEEF was dissolved in DMSO and diluted with autoclaved distilled water.

### Phytochemical investigation

Phytochemical examination indicated significant amount of flavonoids (~44.7 mg/g^DW^), saponins (~42.1 mg/g^DW^), phenolics (~21.3 mg/g^DW^), ascorbic acid (~2.6 mg/g^DW^) and carbohydrates (~112.2 μg/g^DW^) in AEEF as we described in our previous report [10]. RP-HPLC (Dionex Ultimate 3000 HPLC system, Dionex, Germany) examination indicated presence of quercetin, myricetin, gallic acid and chlorogenic acid in AEEF as we mentioned in our previous report [10].

### Animals

Healthy ♂ Swiss albino mice (22 ± 2 g) were used in this study. The mice were housed under standard conditionsof light–dark cycle (12 h), relative humidity (50 ± 15%), temperature (25 ± 2°C), water and standard diet *ad libitum*. The values of laboratory animal care were followed throughout the experiment [15]. The animal ethics committee (Ref. no. 0367/01/C/cpcsea), Jadavpur University approved (Ref no. AEC/PHARM/1501/02/2015 dated 18.03.2015) this experiment with animals.

### *In vitro* bio-assays

#### Hepatocyte isolation and assessment of cytoprotective role of AEEF

Hepatocytes were obtained from mouse liver by previously described method [10]. The hepatocytes were passaged a couple of times to achieve ~ 100% viable cells for the *in vitro* assays. The toxic effect of Pb-acetate was demonstrated in our earlier investigation employing MTT assay [6]. Pb-acetate exhibited IC_50_ value of 6.8 μM in murine hepatocytes. Cell viability test has been conducted to assess the protective effect of AEEF against Pb-acetate induced cytotoxicity. Various sets of hepatocytes (~2×10^6^ cells/set) were exposed to Pb-acetate (6.8 μM) and Pb-acetate (6.8 μM) along with AEEF (50, 100, 200 and 400 μg/ml). The cell viabilities were measured employing MTT assay at different intervals up to 4 h [16]. One set of hepatocytes with Pb-acetate was used as toxic control, while, an untreated set of hepatocytes was used as normal control.

#### Hoechst staining

Hoechst staining was done to observe fluorescent nuclei as well the nuclear pattern within the viable cells [17]. Briefly, the fixed-hepatocytes were exposed to Hoechst 33258 (5 μg/ml in PBS) for 15 min followed by washing with PBS. Fluorescent nuclei were counted as a key of cell viability. One set of hepatocytes with Pb-acetate was kept as toxic control, while, an untreated set of hepatocytes was kept as normal control.

#### Assays of antioxidant markers

Different sets of hepatocytes containing suspension (1 ml; ~ 2×10^6^ cells/ml) were exposed to Pb-acetate (6.8 μM) + AEEF (400 μg/ml) at 37°C for 2 h [16]. One set of hepatocytes treated with Pb-acetate (6.8 μM) was served astoxic control. A set of untreated hepatocytes was kept for serving as normal control. Production of intra-cellular ROS was calculated by measuring DCF-formation in a fluorescence spectrometer (Olympus-1X70, Japan, software-Metamorph) [18,19]. The amount of lipid peroxidation was evaluated by estimating thiobarbituric acid reactive substances (TBARS), a by-product of lipid peroxidation [20]. The extent of protein carbonylation was measured by the Uchida and Stadtman’s protocol [21]. The levels of endogenous antioxidant enzymes namely catalase (CAT), superoxide dismutase (SOD), glutathione peroxidase (GPx), glutathione reductase (GR) and glutathione-S-transferase (GST) were estimated spectrophotometrically [22]. Reduced glutathione (GSH) level was quantified by the method developed by Hissin and Hilf [23].

#### Immunoblotting of signaling proteins

Sample proteins (10 μg) isolated from the hepatocytes under different treatments were subjected to SDS-PAGE (10%) and were transferred into nitrocellulose membranes [24]. Membranes were blocked (4°C; 1 h) in blocking buffer containing non-fat dry milk (5%) in tris-buffered saline containing 0.1% Tween-20 (TBST) and then washed (5 min/washing) with TBST. Later, the said membranes were incubated with primary antibodies viz. anti-caspase 3,8,9 (1: 1,000 dilution), anti-Bad (1: 2,000), anti-Bcl-2 (1: 2,000 dilution), anti-Fas (1: 1,000), anti-Bid (1: 1,000), anti-cyt C (1: 1,000), anti-Apaf-1(1: 1,000) at 4°C overnight followed byrinsing with TBST. The membranes were then exposed to suitable HRP conjugated secondary antibody (1: 3,000) at room temperature for 1 h. The blots werefinally established by 3,3′-diaminobenzidine tetrahydrochloride. The membranes were then subjected to mild stripping in stripping buffer containing 1% SDS (pH 2.0) and glycine (25 mM) followed by application of anti-β actin (1: 6,000) primary antibody at 4°C overnight. The membranes were then washed with TBST followed by secondary antibody treatment and detection as described before.

### *In vivo* bioassay

#### Experimental design

After 2 weeks of intimacy with laboratory/experimental atmosphere, the experimental Swiss Albino mice (n = 18) were divided into 3 groups (6/group). A group (Group I) of animals was treated with double distilled water for 40 days and served as normal control. The animals under toxic control (Group II) group were treated with the aqueous solution of Pb-acetate (5 mg/kg, orally) for 40 days [3]. The mice under Group III received AEEF (100 mg/kg, orally) once daily for 30 days prior to Pb-acetate (5 mg/kg, orally) administration from day 11 onward after start of Pb-acetate treatment [3]. The condition of the animals was monitored twice daily throughout the course of this study to see any behavioral abnormality. The dose of Pb-acetate (< 1/100^th^ of LD_50_ of Pb-acetate) has been chosen on the basis of previously described protocols [3, 6]. No animal died during the period of the experiment.

**Fig 1:**
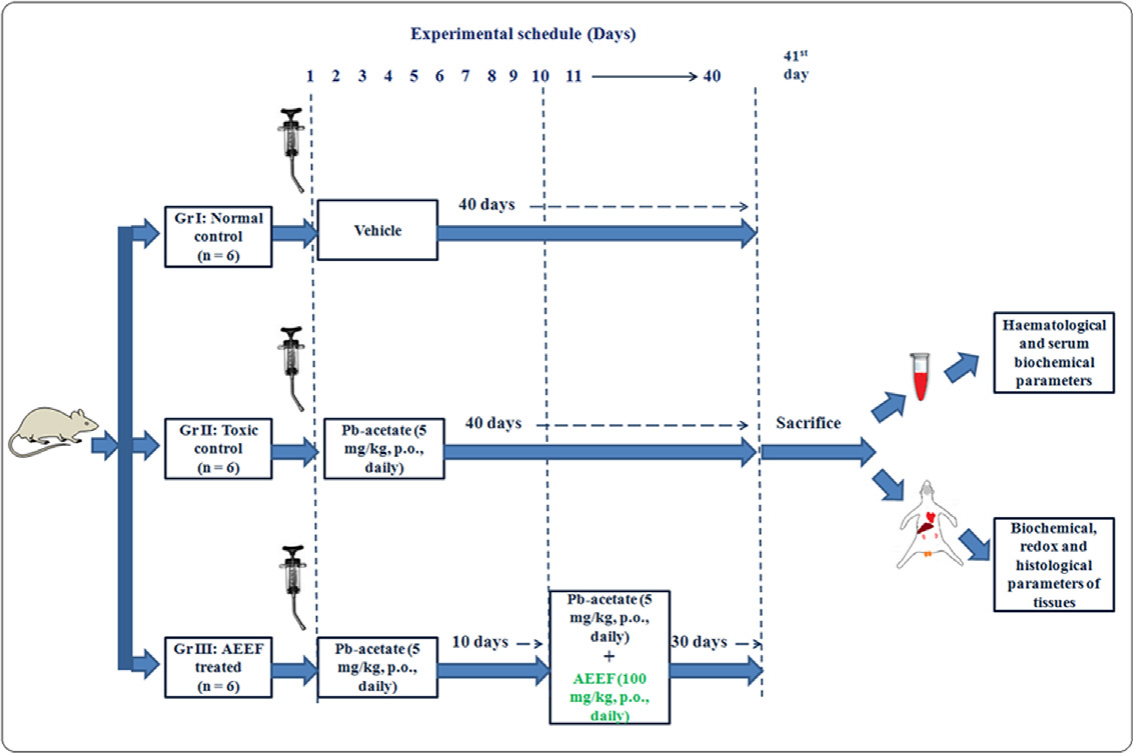
A Schematic overview of *in vivo* experimental protocol.

After 40 days of treatment, the mice were exposed to CO_2_ euthanasia and blood samples were obtained from retro-orbital venous complex. The blood sample was collected without anesthetizing the animals as per recommendation. The mice were then sacrificed by cervical dislocation. The organs were excised and washed with pH 7.4 PBS (phosphate buffer saline) to remove adhering blood and other unwanted debris/fluids. The organs were mixed in Tris-HCl- EDTA (0.1 M: 0.001 M) buffer of pH 7.4 and centrifuged (12,000 g) at 4°C for ½ h. The supernatantsobtained were used for the subsequent assays. A schematic view of overall *in vivo* assay has been depicted in Fig 1.

#### Haematological parameters

The erythrocytes and total leucocytes were counted using a haemocytometer. The haemoglobin content was measured by a haemoglobinometer. The LDH, CK, total cholesterol and triglycerides contents were measured by kit (Span Diagnostic Limited, India) methods.

#### Assessment of antioxidant markers related to organ dysfunction

Presence of Pb within the tissues was quantified by atomic (flame) absorption spectrophotometer. The extent of ROS occurrence, peroxidation of lipids, carbonylation of proteins, endogenous antioxidant enzymes, and non-enzymatic antioxidants were evaluated by previously described standard methods. Quantities of fragmented DNA were measured by the colorimetric diphenylamine reaction [25]. DNA oxidation was measured by HPLC and represented as the ratio of 8-OHdG to 2-dG [26]. Intracellular ATP concentrations were assessed following protocol mentioned in the kit (Abcam, Cambridge, USA). The levels of Co-enzymes Q_9_ and Q_10_ within the tissue extract were measured by the method of Zhang et al [27].

#### Histopathological studies

Formalin (10%) was used to fix the organs of mice immediately after sacrificing. The formalin fixed organs were embedded within paraffin blocks. The paraffin blocks were processed for microtome sectioning. Hematoxylin-Eosin (H&E) staining of sections (5 μm) was done for histological assessments [28]. Histo-quantifications were achieved employing NIH IMAGE (Image-J, 1.37v) software. 60 randomly selected plates/group was investigated to obtain histo-quantification data [29–35].

### Statistical analysis

The experimental data were statistically interpreted by one-way ANOVA and articulated as mean ± SE followed by Dunnett’s t-test using computerized GraphPad InStat (version 3.05), GraphPad software, USA. The values were considered significant when p < 0.05.

## Results

### Effect of AEEF against Pb-acetate-intoxication *in vitro*

#### Effect on cell viability

**Fig 2:**
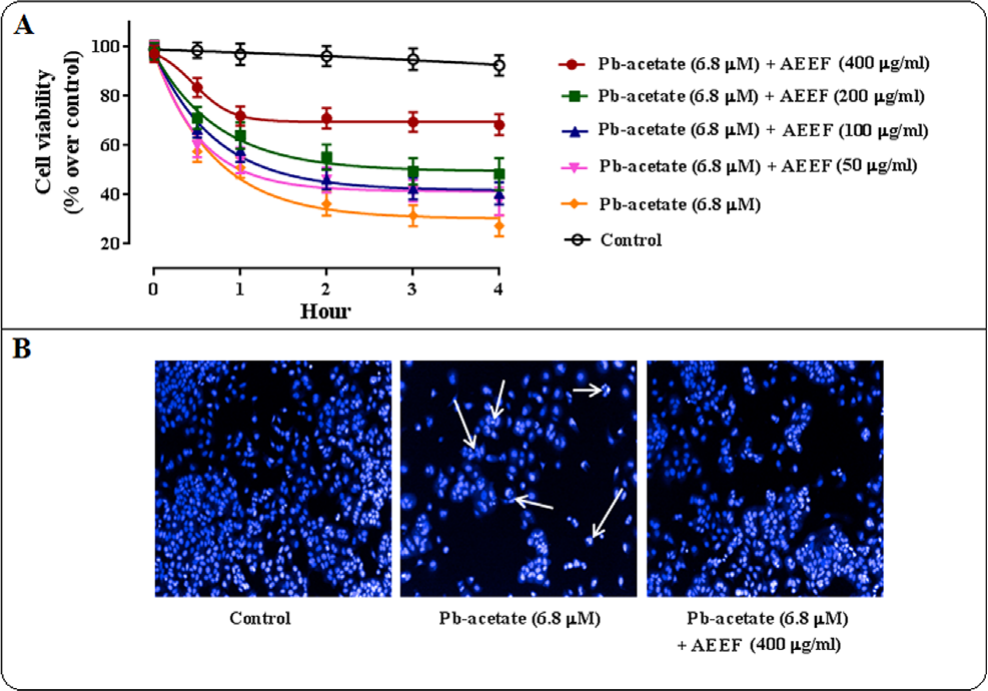
The effect on cell viability assay in the absence (Pb-acetate) and presence of AEEF (Pb-acetate + AEEF) in isolated murine hepatocytes. Panel A. Effect on cell viability assayed in the absence (Pb-acetate) and presence of AEEF (Pb-acetate + AEEF). Panel B. Hoechst staining of mouse hepatocytes in the absence (Pb-acetate) and presence of AEEF (Pb-acetate + AEEF). The white arrows represented the heterogeneous intensities and chromatin condensation of nuclei. Results were represented as mean ± SE (n = 3).

The cell viability was measured after incubating the hepatocytes with AEEF (50–400 μg/ml) along with Pb-acetate (Fig 2A). Pb-acetate treatment caused a gradual decrease (~98–28%) in the viability of murine hepatocytes up to 4 h. The restoration of cell viability indicated the cytoprotective effect of AEEF in a concentration dependent manner. AEEF @ 400 μg/ml exhibited the best cytoprotective effect and restored the cell viability ~ 68%. However, the untreated hepatocytes maintained the cell viability almost linearly (~99–93%).

To observe the cytoprotective effect of AEEF, an image assay was performed using Hoechst stain (Fig 2B). Hoechst staining of Pb-acetate treated hepatocytes indicated notably the low number of visible nuclei as compared with untreated hepatocytes. Besides, Pb-acetate treated hepatocytes exhibited heterogeneous intensity and chromatin condensation of nuclei. On other hand, AEEF treatment could significantly restore the viability of hepatocytes evident from nuclear count.

#### Effect on ROS-generation, redox status, protein-carbonylation and lipid-peroxidation

**Fig 3:**
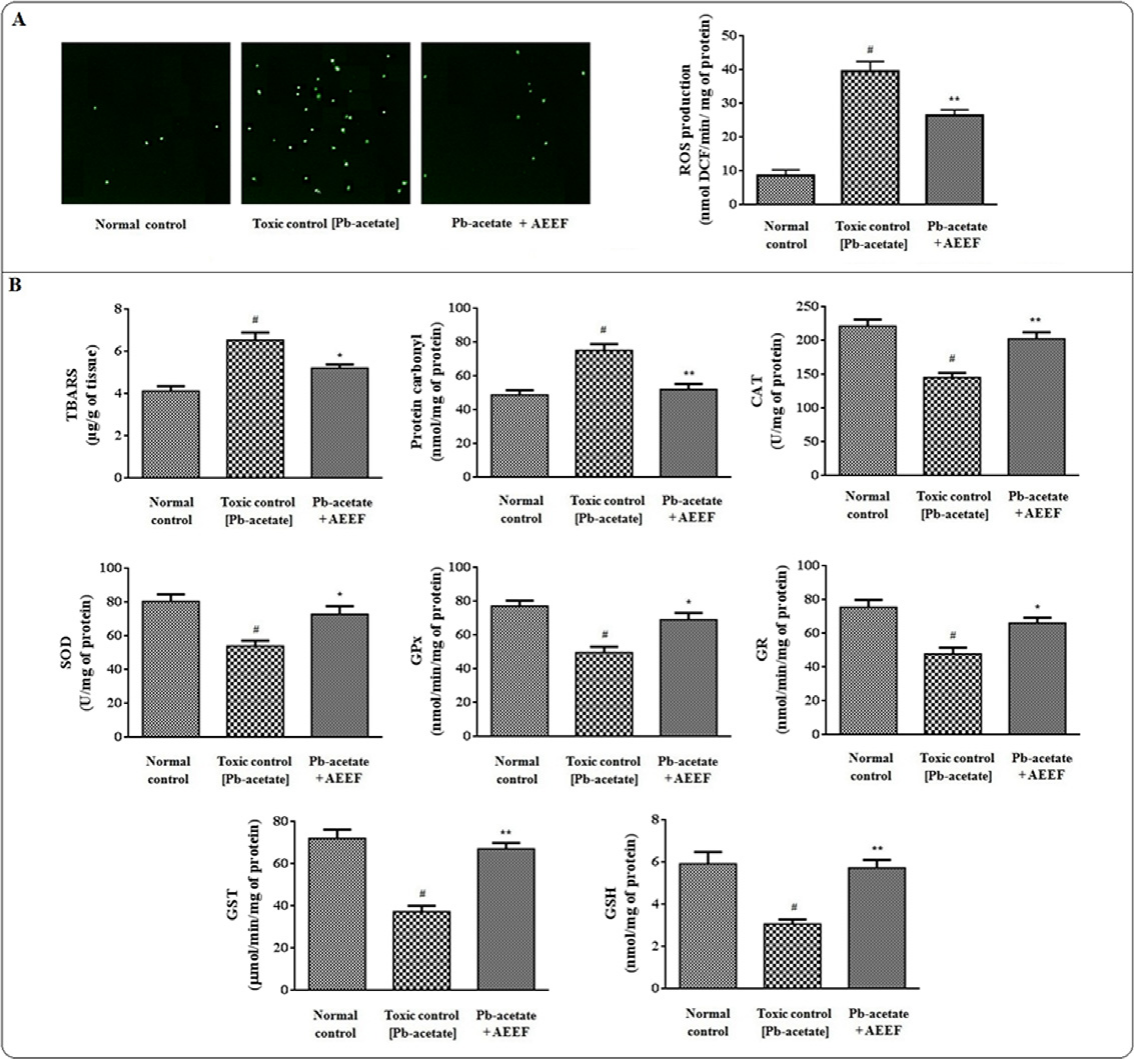
The effect on cellular ROS production, degree of lipid peroxidation, protein carbonylation, endogenous redox systems in the absence (Pb-acetate) and presence of AEEF (Pb-acetate + AEEF) in isolated mouse hepatocytes. Panel A. Effect on ROS-generation in Pb-exposed hepatocytes was measured by fluorescence microscopy in the absence (Pb-acetate) and presence of AEEF (Pb-acetate + AEEF). Panel B. Effect on protein carbonylation, lipid peroxidation and endogenous redox status in the absence (Pb-acetate) and existence of AEEF (Pb-acetate + AEEF). Results were represented as mean ± SE (n = 3). ^#^Results were significantly (p < 0.01) different from normal control. *Results were significantly (p < 0.05) different from Pb-acetate control. **Results varied significantly (p < 0.01) from Pb-acetate control. SOD unit, “U” was defined as inhibition (μ-moles) of NBT-reduction/min while CAT unit “U” was defined as H_2_O_2_ consumed/minute.

ROS production is an indication of redox-challenged cellular environment. In presence of ROS, non-fluorescent DCFH (generated from deacetylated DCFH-DA in presence of viable cells) quantitatively reacted with free radicals to form DCF which has been measured by fluorescence microscopy. Pb-acetate treatment increased significantly (p < 0.01) the production of intracellular ROS within the hepatocytes *in vitro* (Fig 3A). However, AEEF treatment significantly counteracted with ROS generation within Pb-exposed hepatocytes. ROS directly attacks cellular macromolecules, which results in the peroxydative damage of membrane lipids and carbonylation of cellular proteins. The degree of lipid peroxydation was estimated by measuring the TBARS levels. In this study, Pb-acetate exposure could significantly (p < 0.01) elevate the TBARS level in isolated murine hepatocytes (Fig 3B). In search of proteins’ carbonylation, Pb-acetate treated hepatocytes showed significantly (p < 0.01) higher level of carbonylated proteins (Fig 3B). AEEF treatment, however, could significantly alleviate the protein carbonylation (p < 0.01) and lipid peroxydation (p < 0.05) as compared to Pb-intoxicated hepatocytes. Endogenous redox systems viz. cellular antioxidant enzymes and reduced glutathione are the potential targets of Pb-poisoning. In this study, a significant (p < 0.01) depletion of cellular antioxidant enzymes (CAT, SOD, GR, GPx and GST) and GSH levels in Pb-exposed hepatocytes corroborated the Pb-mediated oxidative challenges within the cells (Fig 3B). However, AEEF treatment significantly (p < 0.05–0.01) attenuated the Pb-acetate mediated down regulation of antioxidant enzymes and GSH within murine hepatocytes as compared to Pb-exposed hepatocytes.

### Effects on apoptotic events

**Fig 4:**
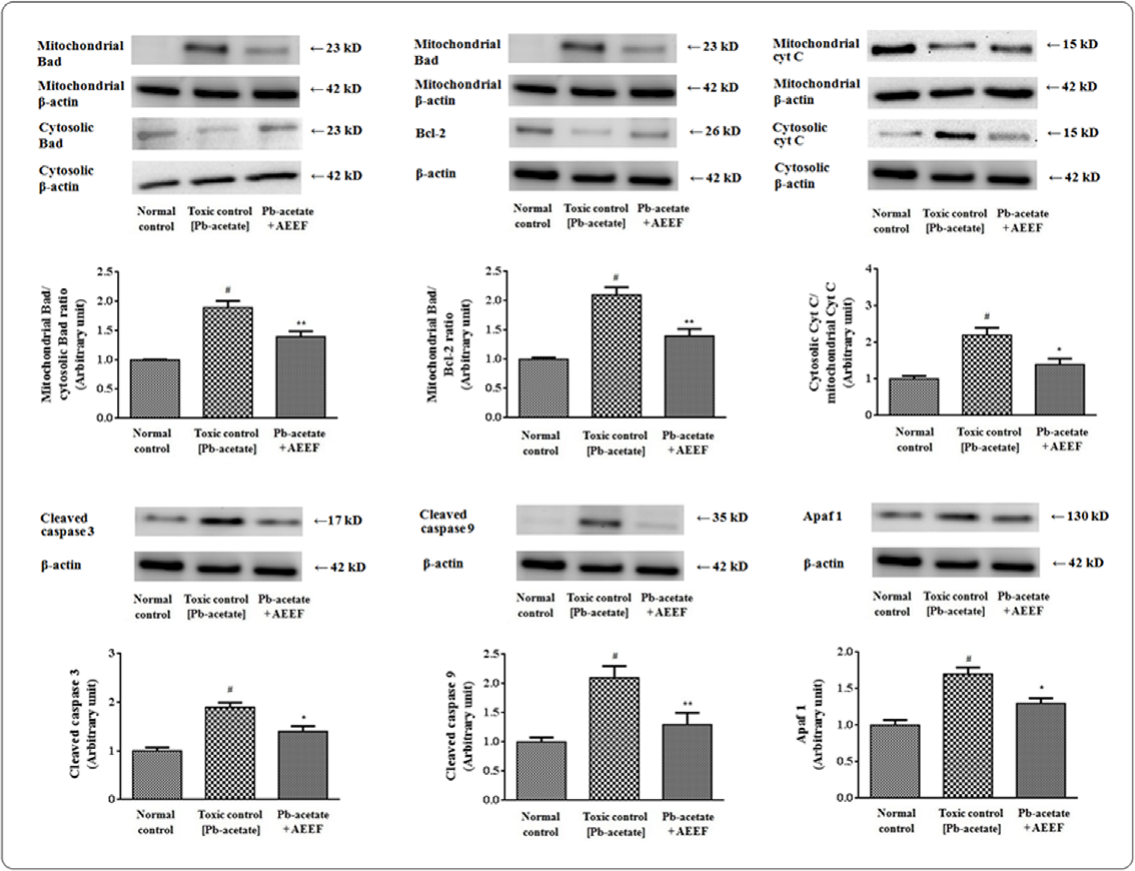
Immunoblot analysis of intrinsic factors of apoptotic event in the absence (Pb-acetate) and presence of AEEF (Pb-acetate + AEEF) in isolated murine hepatocytes. The comparative band intensities were evaluated and the normal control band was assigned a random value of 1 [The ratio of the band intensity of signal protein under normal control and the band intensity of β actin under normal control group was unified and subsequently the band intensities of other groups were obtained by multiplying with same factor used during unification of the protein expression in normal control group]. Loading protein used was β actin. Results were expressed as mean ± SE (n = 3). ^#^Values significantly (p < 0.01) differed from normal control. *Results significantly (p < 0.05) differed from Pb-acetate. ** Results significantly (p < 0.01) differed from Pb-acetate control.

**Fig 5:**
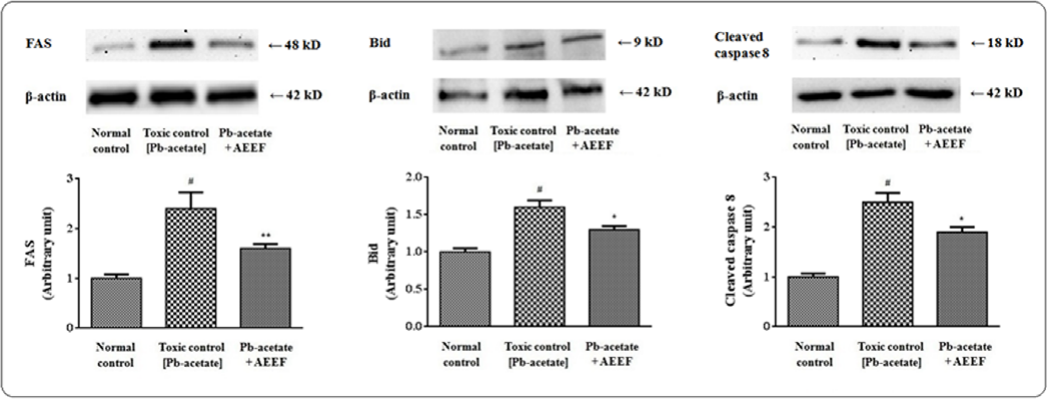
Immunoblot analysis of extrinsic factors of apoptotic event in the absence (Pb-acetate) and existence of AEEF (Pb-acetate + AEEF) in isolated mouse hepatocytes. The relative band intensities were quantified and the normal control band was assigned an arbitrary value of 1. Loading protein used was β actin. Results were expressed as mean ± SE (n = 3). ^#^Values significantly (p < 0.01) differed from normal control. *Results significantly (p < 0.05) differed from Pb-acetate. ** Results significantly (p < 0.01) differed from Pb-acetate control.

Oxidative stress can trigger the apoptotic events by reciprocating the transcriptions of different apoptotic proteins. In this study, the expressions of different apoptotic factors involved in mitochondria dependent and independent apoptotic events were evaluated employing western blot technique (Figs 4 and 5, respectively). Pb-acetate treated murine hepatocytes exhibited a significant movement of Bad protein to mitochondria from cytosol evident from a significantly (p < 0.01) high mitochondrial/cytosolic Bad ratio as compared to normal control hepatocytes (Fig 4). A significant ↑-regulation of mitochondrial Bad protein with simultaneous ↓-regulation of Bcl-2 resulted a significantly (p < 0.01) high mitochondrial Bad/cellular Bcl-2 ratio (Fig 4). The later instigate the significant (p < 0.01) translocation of Cyt C from mitochondria into cytosol (Fig 4). The discharge of Cyt C into cytosol promotes cleavage of caspases (caspase 3 and 9) into their respective slashed (active) fractions. In current investigation, Pb-intoxicated hepatocytes exhibited a significant (p < 0.01) ↑-regulation of the expressions of cleaved fractions of caspase 3 and 9. The cytosolic Cyt C binds with Apaf 1 to assist the formation of apoptosomes. A significant ↑-regulation of Apaf 1 was observed in Pb-exposed hepatocytes. From the aforementioned observation suggested that the involvement if mitochondria dependent (intrinsic) pathway of apoptosis following Pb-intoxication in the murine hepatocytes (Fig 4). However, AEEF treatment significantly (p <0.05–0.01) reinstated the Pb-mediated changes in the expressions of intrinsic apoptotic proteins near to normalcy (Fig 4).

In search of effect of Pb-acetate on mitochondria independent (extrinsic) apoptotic pathway and protective role of AEEF, immunoblottings were performed (Fig 5). Western blot analysis of Bid, FAS and cleaved caspase 8 revealed a significant (p < 0.01) ↑-regulation of the transcriptions of extrinsic apoptotic proteins in the mouse hepatocytes following Pb-intoxication. However, AEEF treatment reduced significantly (p < 0.05–0.01) the expressions of cleaved caspase 8, Bid and FAS (Fig 5) and thereby inhibited the extrinsic pathway of apoptosis.

#### Blood parameters

The effects of various treatments on blood parameters were revealed in Table 1. Pb-acetate treated mice exhibited significantly (p < 0.01) elevated blood Pb-content. Amongst haematological parameters, Pb-intoxication significantly (p < 0.01) reduced erythrocytes content with concomitant reduction (p < 0.01) of haemoglobin level. However, no significant effect was observed in total leucocytes content. Pb-acetate treatment significantly (p < 0.01) enhanced the serum LDH, CK, total cholesterol and triglycerides levels. However, AEEF treatment significantly (p < 0.05–0.01) reinstated Pb-acetate mediated abnormalities of serum biochemical and haematological parameters near to normal levels.

**Table 1:** Effect on blood parameters in the absence (Pb-acetate) and presence of AEEF (AEEF + Pb-acetate) in mice.

Groups	Serum biochemical and haematological parameters	Values
Normal control	Pb content (μg/ml)	0.05 ± 0.004
Toxic control (Pb-acetate)		0.87 ± 0.05^#^
Pb-acetate + AEEF		0.69 ± 0.05*
Normal control	Total erythrocytes count (x10^6/^mm^3^)	5.50 ± 0.29
Toxic control (Pb-acetate)		3.32 ± 0.45^$^
Pb-acetate + AEEF		4.56 ± 0.32
Normal control	Haemoglobin (g/dl)	9.23 ± 0.48
Toxic control (Pb-acetate)		6.04 ± 0.76^#^
Pb-acetate + AEEF		8.05 ± 0.32*
Normal control	Total leucocytes count (x10^3/^mm^3^)	5.67 ± 0.21
Toxic control (Pb-acetate)		5.58 ± 0.72
Pb-acetate + AEEF		5.78 ± 0.32
Normal control	LDH (U/l)	33.18 ± 1.57
Toxic control (Pb-acetate)		53.04 ± 2.34^#^
Pb-acetate + AEEF		45.05 ± 1.62*
Normal control	CK (IU/ mg protein)	183.33 ± 11.50
Toxic control (Pb-acetate)		254.67 ± 14.45^#^
Pb-acetate + AEEF		201.04 ± 10.23*
Normal control	Cholesterol (mg/dl)	185.41 ± 12.33
Toxic control (Pb-acetate)		264.62 ± 13.98^#^
Pb-acetate + AEEF		212.37 ± 9.77*
Normal control	Triglycerides (mg/dl)	115.22 ± 7.50
Toxic control (Pb-acetate)		194.92 ± 11.54^#^
Pb-acetate + AEEF		123.87 ± 6.59**

Results are expressed as mean ± SE, (6 mice per group).

^$^ Results significantly (p < 0.05) varied from normal control.

^#^ Results varied significantly (p < 0.01) from normal control.

* Results significantly (p < 0.05) varied from Pb-acetate control.

**Results significantly varied (p < 0.01) from Pb-acetate control.

#### Effect on Pb-accumulation, DNA fragmentation, DNA oxidation and ATP levels

**Fig 6:**
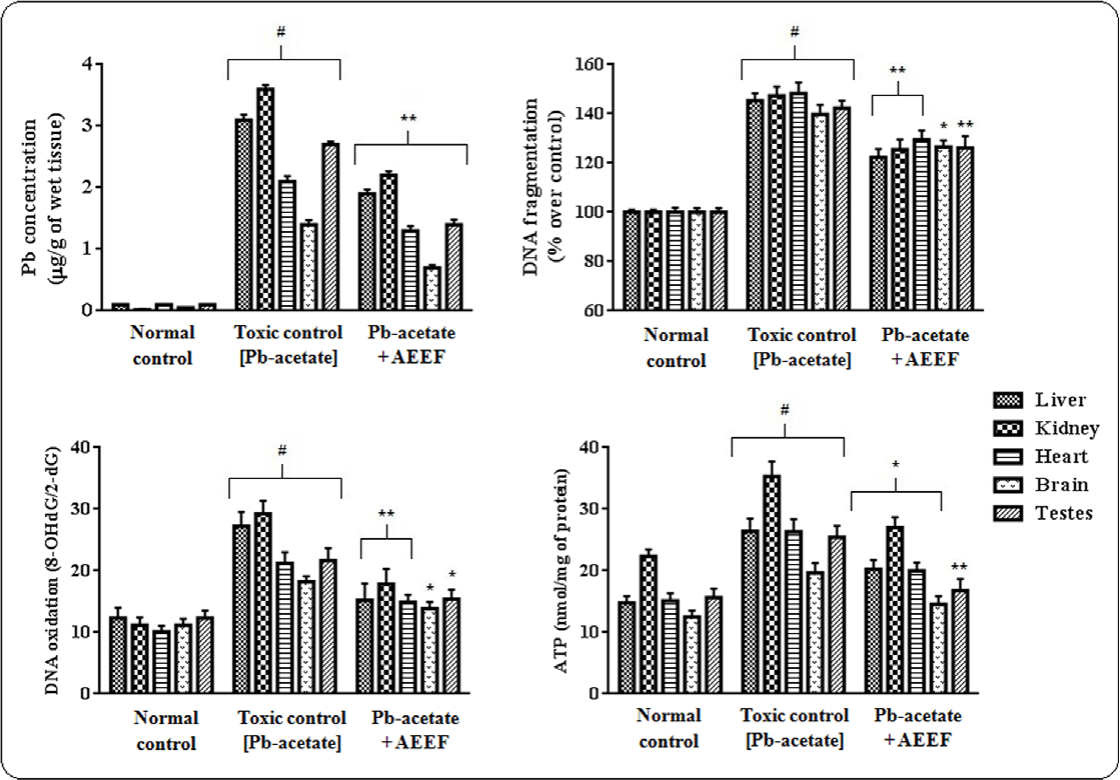
Effect on Pb accumulation (Panel A), DNA fragmentation (Panel B) DNA oxidation (Panel C) and ATP levels (Panel D) in the absence (Pb-acetate) and existence of AEEF (Pb-acetate +AEEF) in heart, kidney, liver, brain and testes in mice. Results were denoted as mean ± SE (n = 6). ^#^Results differed significantly (p < 0.01) from normal control. * Results significantly (p < 0.05) differed from Pb-acetate control. ** Results significantly (p < 0.01) differed from Pb-acetate control.

Like other heavy metals, Pb exerts toxic manifestations after accumulation within the tissues. In this study, Pb-acetate treated mice demonstrated a significantly (p < 0.01) high Pb-burden in the livers, kidneys, hearts, brains and testes (Fig 6). The extent of Pb accumulation has been found to be maximum in kidney, while, the lowest Pb-burden was observed in the brain. AEEF treatment reduced significantly (p < 0.01) the intracellular Pb burden as compared to the respective organs of toxic control animals. In current study, Pb-intoxication significantly (p < 0.01) increased the fragmentation and the oxidation of cellular DNA of the aforementioned organs of experimental mice (Fig 6). On other hand, AEEF treatment attenuated significantly (p < 0.05–0.01) the DNA fragmentation and oxidation. The DNA-protective effect of AEEF could be endorsed with the overall cytoprotective effect AEEF. The cellular ATP concentration gives a primary idea about the incidence of apoptosis. Significantly (p < 0.01) high values of cellular ATP in the organs of Pb-acetate treated mice vindicated the incidence of apoptotic event within the tissues (Fig 6). However, AEEF treatment reverted significantly (p < 0.05–0.01) the ATP levels within the selected tissues to near normal status.

#### Effect on Protein-carbonylation, lipid peroxidation, co-enzymes Q, ROS, anti-oxidant enzymes and GSH levels

**Fig 7:**
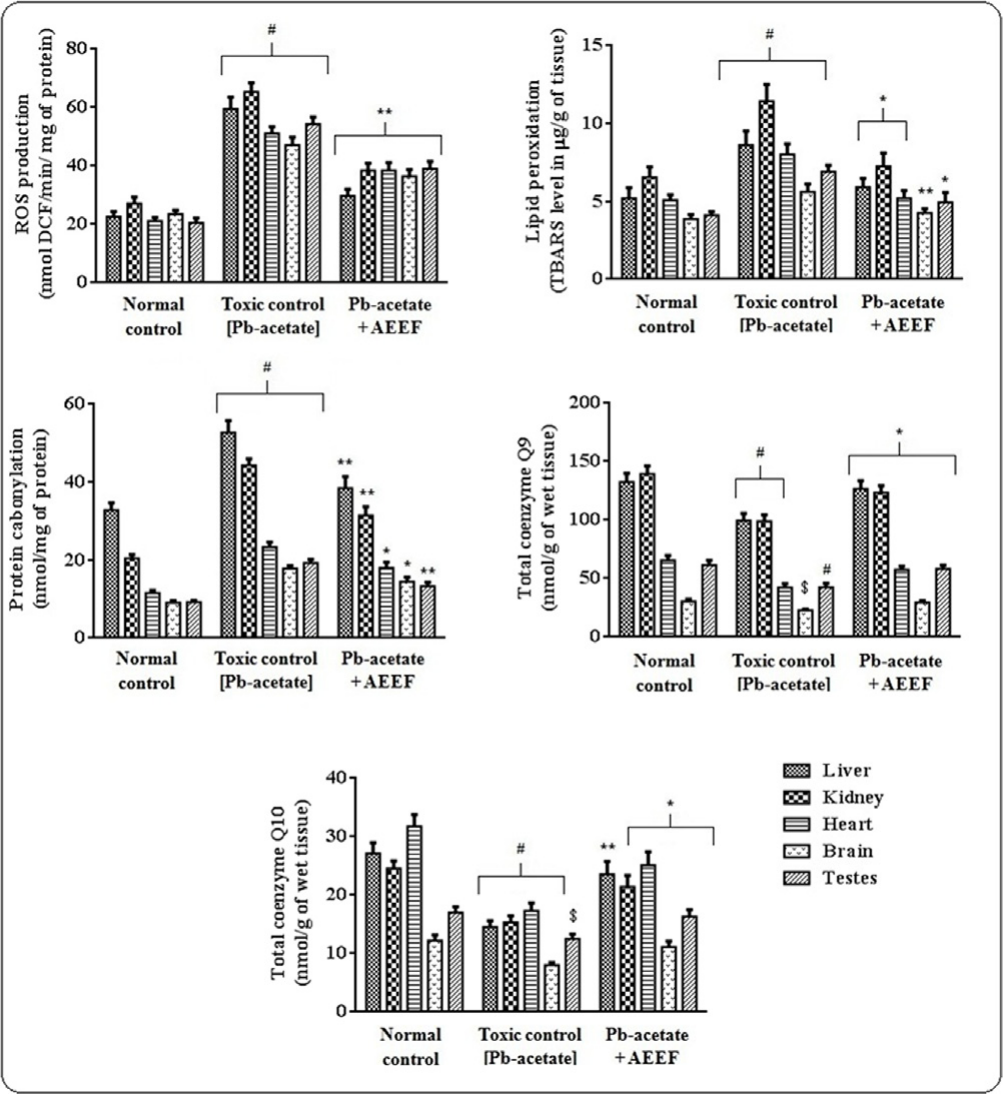
Effect on protein carbonylation, ROS production, lipid peroxidation and co-enzyme Q9 levels in the absence (Pb-acetate) and existence of AEEF (Pb-acetate +AEEF) in experimental mice. Results were represented as mean ± SE (n = 6). ^$^ Results differed significantly (p < 0.05) from Pb-acetate control. ^#^Results differed (p < 0.01) significantly from normal control. * Results significantly (p < 0.05) differed from Pb-acetate control. ** Results differed (p < 0.01) significantly from Pb-acetate control.

**Fig 8:**
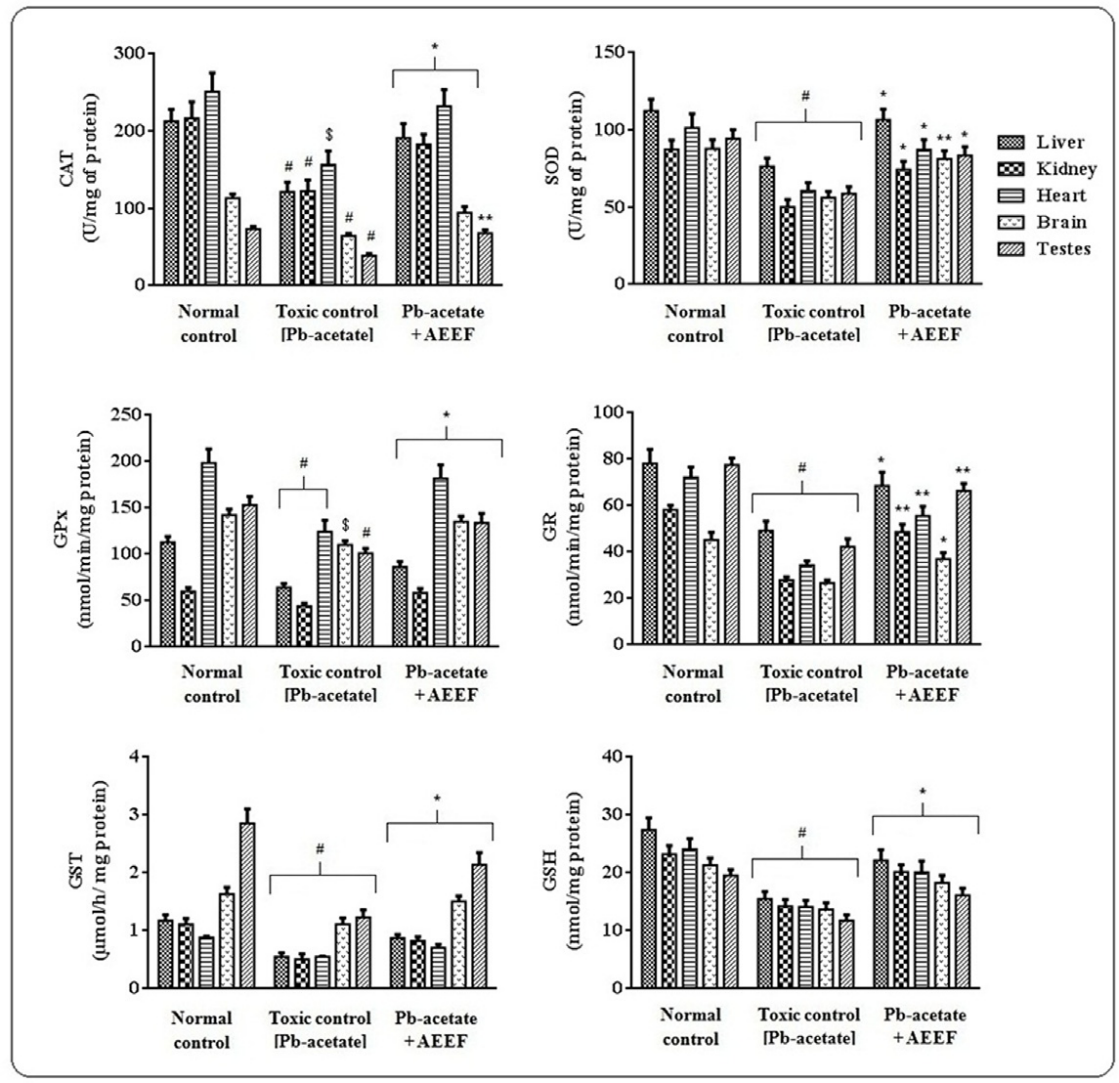
Effect on cellular redox systems in the absence (Pb-acetate) and existence of AEEF (Pb-acetate +AEEF) in experimental mice. Results were represented as mean ± SE (n = 6). ^$^ Results differed significantly (p < 0.05) from Pb-acetate control. ^#^Results differed significantly (p < 0.01) from normal control. * Results significantly (p < 0.05) differed from Pb-acetate control. ** Results significantly (p < 0.01) differed from Pb-acetate control. CAT unit “U” is defined as H_2_O_2_ consumed/minute while SOD unit, “U” is defined as inhibition (μ-moles) of NBT-reduction/min.

In the *in vivo* assay, Pb-acetate treated animals revealed significantly elevated (p < 0.01) levels of intercellular ROS in the tested organs (Fig 7). AEEF treatment, however, significantly (p < 0.01) arrested Pb-acetate mediated ROS generation in the aforementioned tissues. Pb-acetate treatment also could enhance significantly (p < 0.01) lipid peroxidation and protein carbonylation in designated organs of experimental mice, however, AEEF treatment attenuated significantly (p < 0.05–0.01) the carbonylation of proteins and peroxidation of lipids in testicular, cardiac, hepatic, cerebral and renal tissues *in vivo* (Fig 6). Pb-acetate treated mice exhibited significantly (p < 0.05–0.01) lower levels of co-enzyme Q_9_ and Q_10_ in the tissues (Fig 7). Treatment with AEEF significantly (p < 0.05–0.01) reinstated this Pb-induced alteration of co-enzymes Q levels in heart, testes, kidney, brain and liver of experimental mice. Fig 8 depicted the effect on antioxidant enzymes (CAT, GST, GPx, GR and GST) and GSH, measured from homogenates of the tissues of all experimental mice. Pb-acetate administration significantly reduced the levels of GSH (p < 0.01) and antioxidant enzymes (p < 0.05–0.01) in the testicular, renal, hepatic, cerebral and cardiac tissues. Treatment with AEEF could significantly elevate the tissues GSH (p < 0.05) and antioxidant enzymes (p < 0.05–0.01) levels to near normal status.

#### Effect on histology of the organs

**Fig 9:**
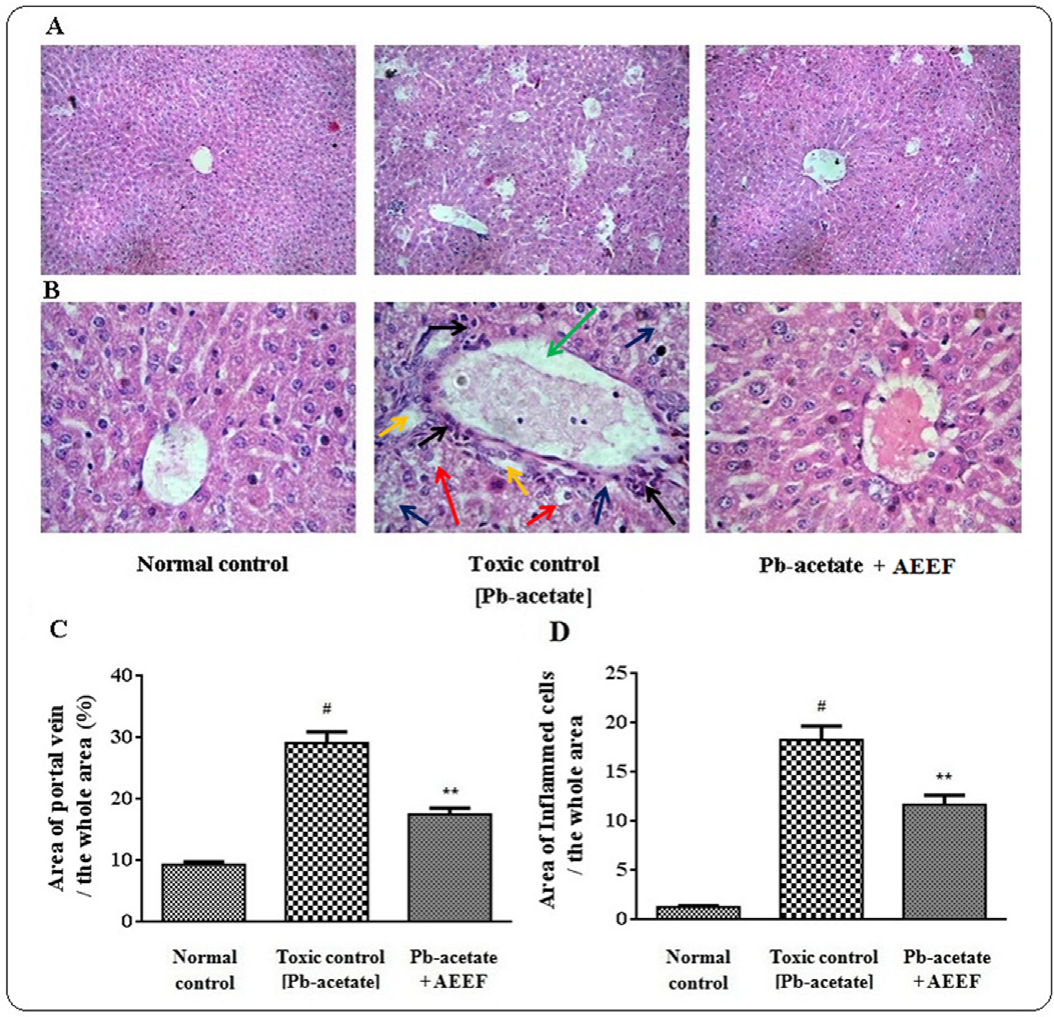
Histological assessments of livers along with histo-quantification data of experimental mice in the absence (Pb-acetate) and existence of AEEF (Pb-acetate + AEEF). Histological sections 100 x (Panel A) and 400 x (Panel B) of livers. The liver section of normal control mice showed normal portal vein and hepatocytes. Pb-acetate treated liver section exhibited dilated portal vein (green arrow), fatty degeneration (blue arrows), vacuolated cytoplasm (red arrows), apoptosis (yellow arrows) and leucocytes infiltration (black arrows) when compared with the section of normal control liver. Panel C. The dilation of portal vein is denoted as % of the blank area comparative to the whole area of the photomicrograph (400 x, arbitrarilynominated areas comprising one portal vein were selected). Panel D. The incidence of inflammation was presented as the % of the inflamed hepatocytes region comparative to the whole area of the photomicrograph (100 x, arbitrarily selected area in portal vein were designated). Values were expressed as mean ± SE, (n = 60). ^#^ Results significantly (p < 0.01)differed from normal control. ^**^Results significantly (p < 0.01) differed from Pb-acetate control.

**Fig 10:**
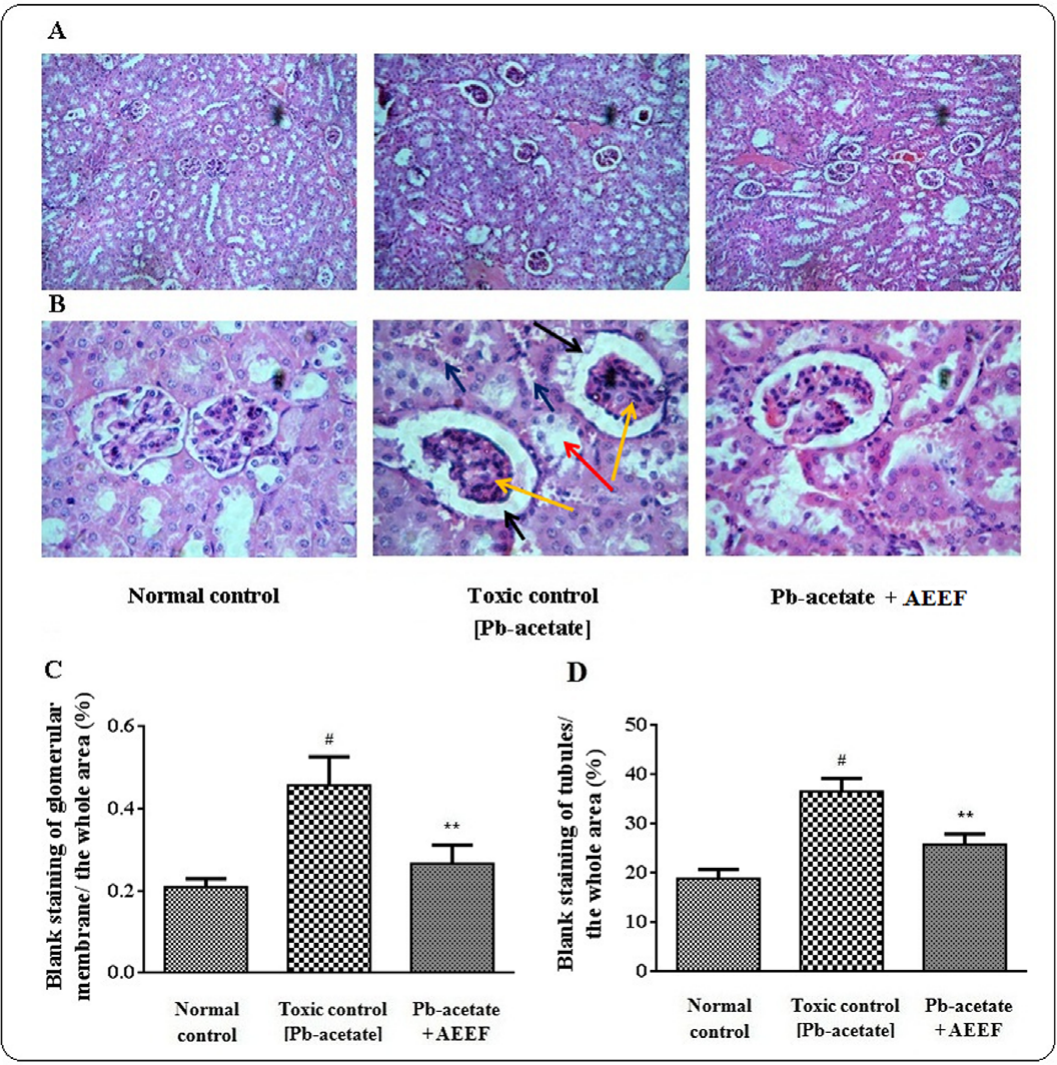
Histological assessments of kidneys along with histo-quantification data of experimental mice in the absence (Pb-acetate) and existence of AEEF (Pb-acetate + AEEF). Histological sections 100 x (Panel A) and 400 x (Panel B) of kidneys. The kidney section of Pb-acetate intoxicated mice exhibited glomerular hypercellularity (yellow arrows), capsular space thickening (black arrows), apoptosis (blue arrows) and cloudy shape of renal tubules (red arrow). Panel C. The broadening of capsular space has been shown as % of the blank area comparative to the whole area of the photomicrograph (400 x, arbitrarily selected areas containing one glomerulus were selected). Panel D. The expansion of tubules has been indicated as the % of the blank area comparative to the whole area of the photomicrograph (100 x, arbitrarilycertain areas devoid of any glomerulus were selected). Results were expressed as mean ± SE, (n = 60). ^#^ Results differed significantly (p < 0.01) from normal control. ^**^Results significantly (p < 0.01) differed from Pb-acetate control.

**Fig 11:**
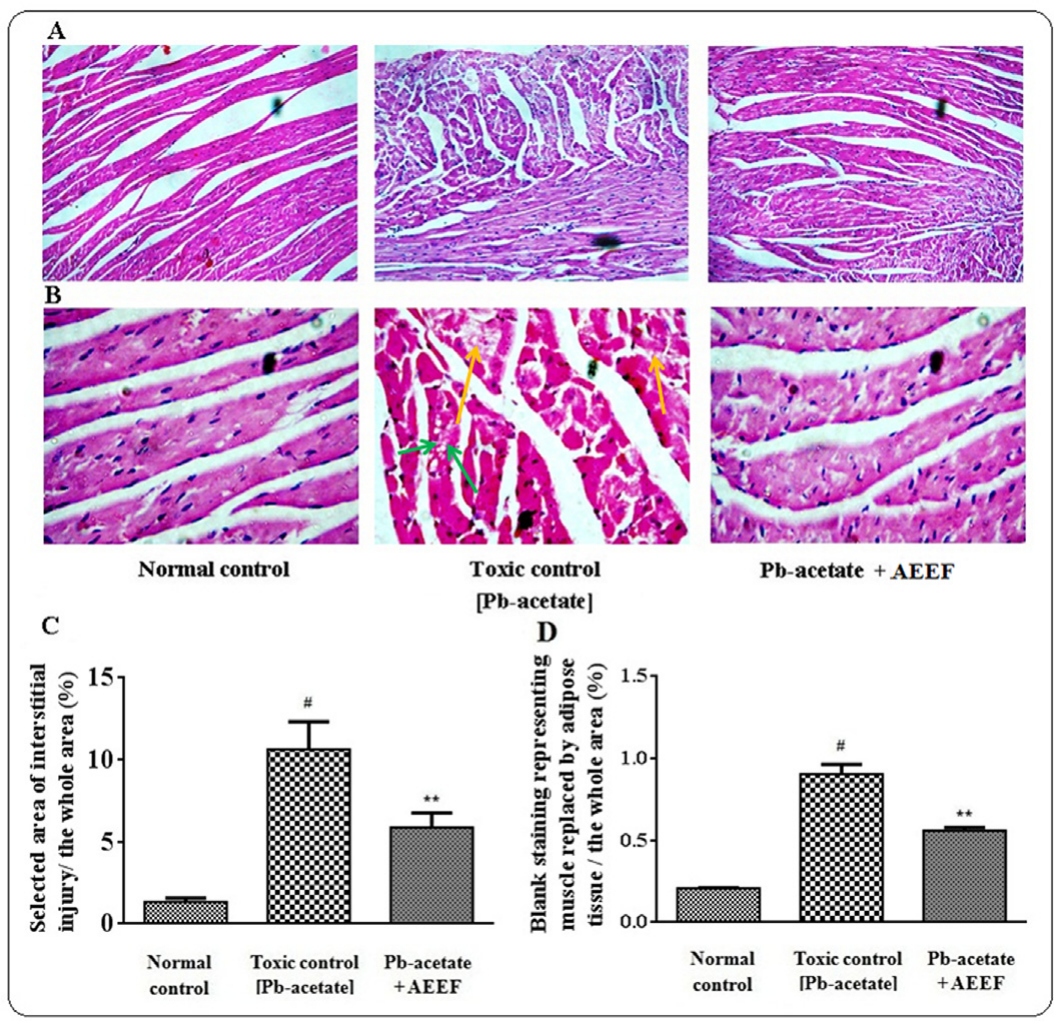
Histological assessments of hearts along with histo-quantification data of experimental mice in the absence (Pb-acetate) and existence of AEEF (Pb-acetate + AEEF). Histological sections 100 x (Panel A) and 400 x (Panel B) of hearts. Pb-acetate intoxicated mice exhibited apoptotic degeneration of interstitial tissues (yellow arrows) and substitution of muscle by adipose tissues (green arrows). Panel C. The manual blank selection of percentage of apoptotic interstitial damagecomparative to the whole area of the photomicrograph (400 x, arbitrarily selected areas) has been represented. Panel D. The percentage of blank designatedpart of adipocyte deposition comparative to the whole zone of the photomicrograph (400 x, arbitrarily selected parts) represented the degree of replacement of tissue by adipocytes. Results were expressed as mean ± SE, (n = 60). ^#^ Results differed significantly(p < 0.01) from normal control. ^**^Results significantly(p < 0.01) differed from Pb-acetate control.

**Fig 12:**
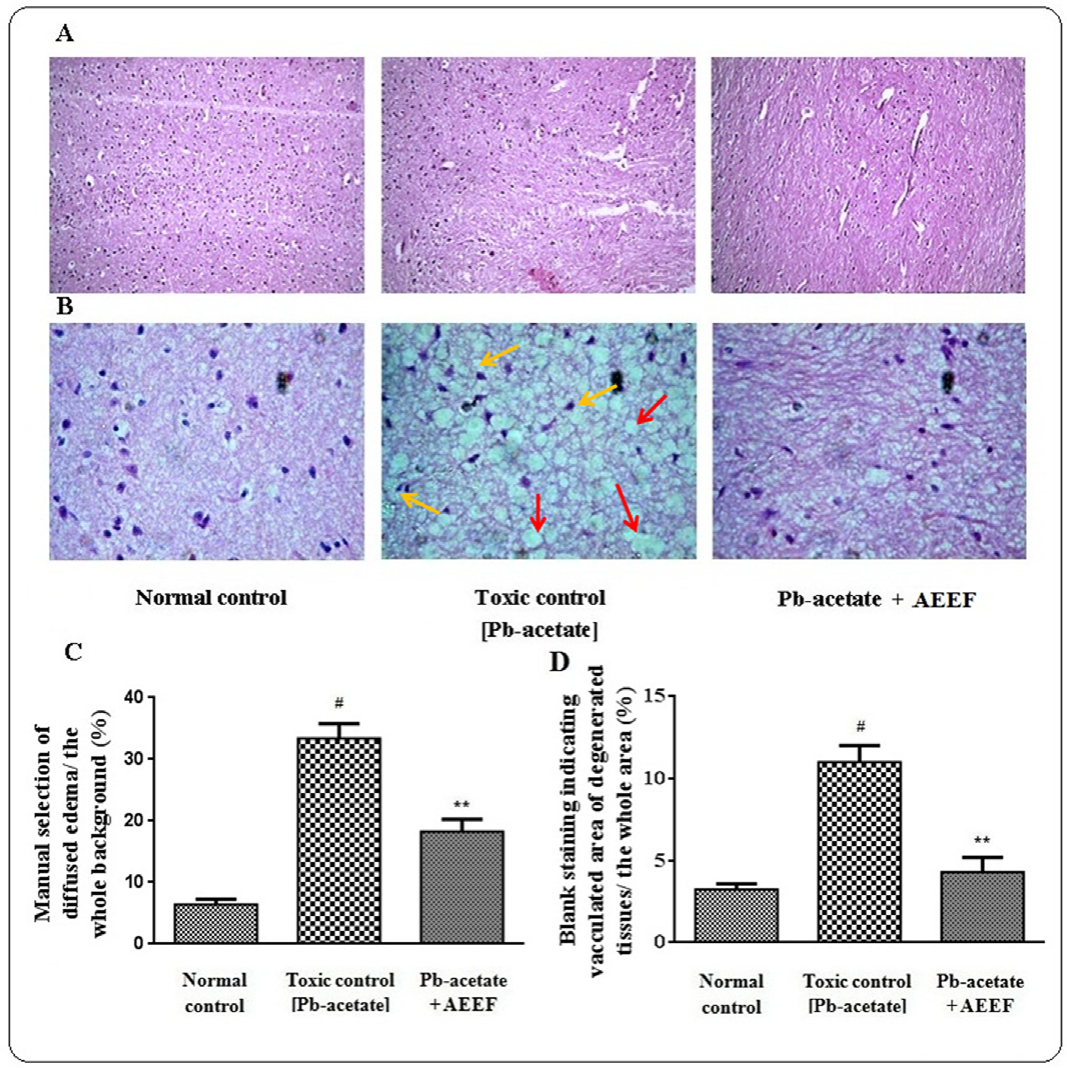
Histological assessments of brains along with histo-quantification data of experimental mice in the absence (Pb-acetate) and existence of AEEF (Pb-acetate + AEEF). Histological sections 100 x (Panel A) and 400 x (Panel B) of brains. Pb-acetate intoxicated mice exhibited apoptotic degeneration of interstitial tissues (yellow arrows) and substitution of muscle by adipose tissues (green arrows). Panel C. The manual blank selection of percentage of apoptotic interstitial damagecomparative to the whole zone of the photomicrograph (400 x, arbitrarily selected parts) has been represented. Panel D. The percentage of blank selected part of adipocyte deposition comparative to the whole zone of the photomicrograph (400 x, arbitrarilyselected parts) represented the degree of substitution of tissue by adipocytes. Results were expressed as mean ± SE, (n = 60). ^#^ Results differed significantly (p < 0.01)from normal control. ^**^Results significantly(p < 0.01) differed from Pb-acetate control.

**Fig 13:**
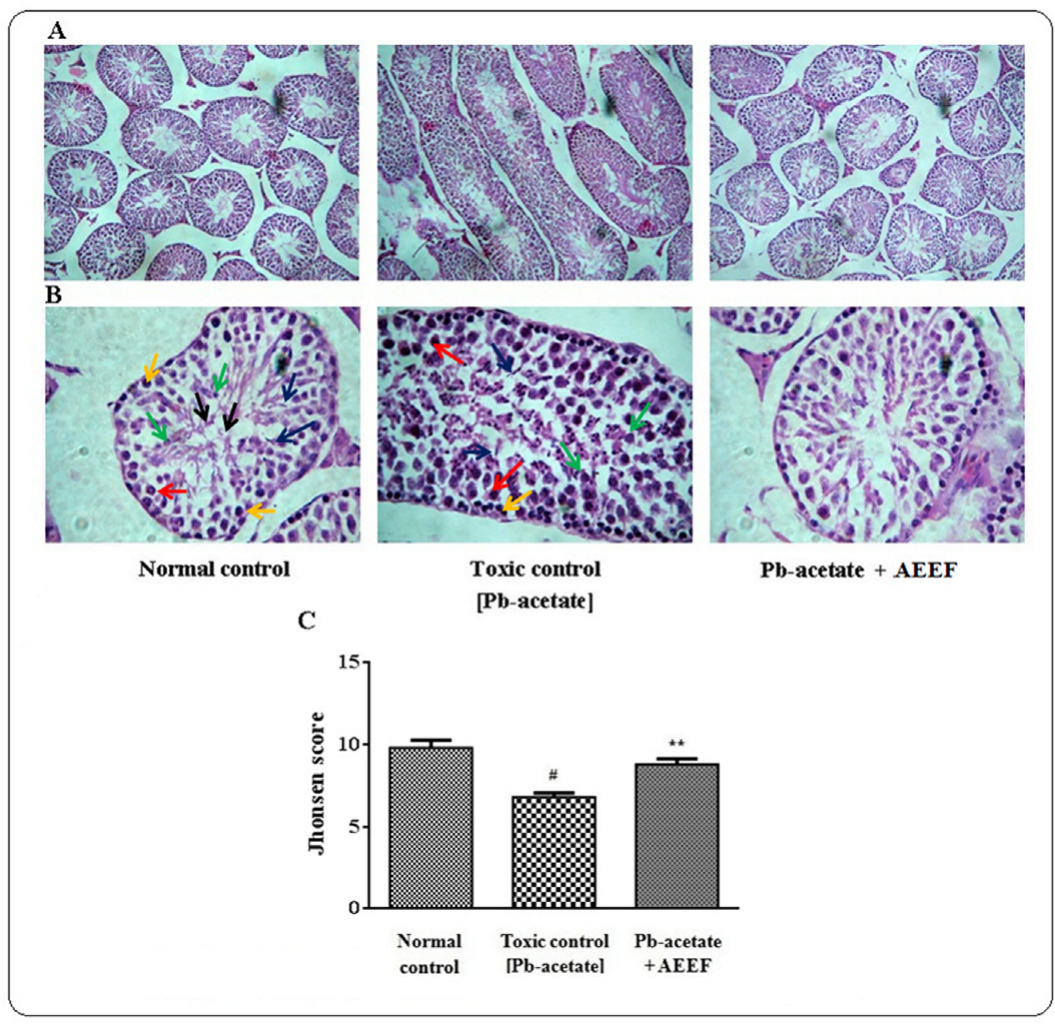
Histological assessments of testes along with histo-quantification data of experimental mice in the absence (Pb-acetate) and existence of AEEF (Pb-acetate + AEEF). Histological sections 100 x (Panel A) and 400 x (Panel B) of testes. The section of testes of normal control mice exhibited all stages of spermatogenesis, while, testes of Pb-acetate treated mice showed disruption of normal arrangement of seminiferous tubules also in the process of spermagenesis. Yellow arrows represented spermatogonia close to the basement membrane; red arrow represented primary spermatocytes; green arrows represented round spermatids; blue arrows denote elongated spermatids; black arrows represented complete spermatozoa. However, AEEF treatment could attenuate the Pb-acetate mediated toxic manifestations in testes of mice. Panel C. The Johnsen score was measured (400 X, comprising one seminiferous tubule). Seminiferous tubule at Johnsen score 10 presenting all stages of spermatogenesis. The Jhonsen score is descending with the toxic occurrence within testicular tissues. Results were expressed as mean ± SE, (n = 60). ^#^ Results differed significantly (p < 0.01) from normal control. ^**^Results significantly (p < 0.01) differed from Pb-acetate control.

The liver sections of mice under different groups were shown in Fig 9A (x 100) and 9B (x 400) along with histo-quantification data (Fig 9C and 9D). The liver section of Pb-acetate treated mice demonstrated dilated portal vein, fatty degeneration, vacuolization, apoptosisand leucocytes infiltration when compared with the section of normal control liver. Histo-quantification of Pb-intoxicated liver sections indicated a significant (p < 0.01) elevation of % of the area of inflamed hepatocytes and the % of area of portal veins (p < 0.01) (Fig 9C and 9D). AEEF treatment reinstated significantly (p < 0.01) the hepatic inflammation and dilation of portal vein to near normal status. The histological sections of kidneys of mice are shown in Fig 10A (x 100) and 10B (x 400). The kidney section of Pb-intoxicated mice revealed glomerular hyper-cellularity with concomitant widening of capsular space, apoptosis and cloudy damage of renal tubules. An attempt to quantify the histological toxic events, Pb-intoxicated sections exhibited a significant (p < 0.01) widening of capsular space (Fig 10C) and cloudy inflammation of tubules (Fig 10D). However, AEEF treatment could significantly revert the histological abnormalities of renal tissues both qualitatively and quantitatively (p < 0.01) to near normal status. The heart sections Fig 11A (x 100) and 11B (x 400) of Pb-acetate intoxicated animals revealed the abnormal radiating pattern of cardiac muscles with simultaneous damage of interstitial tissues and adipocytes accumulation. The histo-quantification data exhibited significant (p < 0.01) apoptotic damage of interstitial tissues and adipocytes deposition (Fig 11C and 11D). However, treatment with AEEF could significantly (p < 0.01) attenuate the Pb-mediated histological deviations and restore the tissue morphology near to normal condition. The sections of mice brains are shown in Fig 12A (x 100) and 12B (x 400). The qualitative histology of Pb-acetate intoxicated mice exhibited some toxic structural changes like diffused edemaand vacuolated area of deteriorated tissues. Histo-quantification data revealed substantial (p < 0.01) increase in the % diffused edema and the % of the degenerated tissues containing the cells with vacuolated cytoplasm within as compared to normal control animals (Fig 12C and 12D), while, AEEF treated mice showed marked improvement both qualitatively and quantitatively (p < 0.01) to restore the tissue architecture to near normal status. Histological sections of testes have been indicated in Fig 13A (x 100) and 13B (x 400). The testes section of Pb-intoxicated mice revealed structural alteration with concomitant abnormalities in spermatogenesis resulting a substantial (p < 0.01) decrease in Jhonsen score (13C). AEEF treatment could offer significant (p < 0.01) reversal of toxic manifestation of Pb-acetate.

## Discussion

Pb, probably the oldest metal, has been recognized as a major risk for the people of developing countries. The wide range of commercial applicability has led to a manifold ascend in the distribution of Pb including in the living systems. The exact mechanism of Pb-toxicity is not quite obvious, however, Pb-mediated generation of excessive ROS and disturbances of endogenous antioxidants have been proposed to participate in the overall toxic manifestations of Pb. Oxidative stress further contribute in the oxidative damage of structural and functional biomolecules and apoptotic damage of the tissues [24,36]. The current study describes the protective role of aqueous extract *E*. *fluctuans* against Pb-toxicity with the help of suitable *in vitro* and *in vivo* bio-assays. Special care has been taken to evaluate the mechanism of protection and to integrate the observed pharmacology with phytochemistry.

ROS production is believed to be the key mechanism of Pb-toxicity. The experimental observation revealed that Pb-acetate exposure could significantly up-regulate intracellular ROS production both *in vitro* and *in vivo*. Increase cellular uptake of Pb would be directly responsible for this ROS generation. Enhanced accumulation of Pb within the tissues within the system following Pb-acetate treatment has been shown. Excessive generation of ROS also directly caused oxidative damage of membrane lipid, cellular proteins and enzymes and nucleic acids [37, 38]. A significant increase in TBARS content, carbonylated proteins and DNA fragmentation and oxidation could be correlated with enhanced ROS production following Pb-acetate treatment. However, AEEF treatment could significantly attenuate the aforementioned ROS mediated pathogenesis of cellular macromolecules. The effect might be due to the inhibition of ROS generation by reducing Pb-burden within the selected tissues and/or neutralization of ROS through radical scavenging effect.

Endogenous antioxidant enzymes and thiol-based antioxidants participate integrally in the overall redox defense during redox challenged cellular environment [38–41]. GSH donates H^+^ + e^−^from its -SH groups present in cysteine residues to ROS to neutralize them and convert into GSSG (taking another molecule of GSH). Under normal physiological status, ~ 90% of the total glutathione content exists in form of GSH, while, only ~ 10% is present as GSSG [7]. Pbhas strong likeness toward–SH group, whichdeactivates GSH. Strong affinity of Pb with–SH group also participates in deactivating the endogenous antioxidant enzymes, which, promotes more redox challenged cellular environment via rendering their ineffectiveness to counteract with oxidative free radicals. Pb also directly interacts with the activities of endogenous antioxidant enzymes by inducing the imbalance of other necessary co-factors (cations) required for the catalytic activities of the enzymes [3,7]. In this study, the extract significantly attenuated the Pb-mediated depletion of antioxidant enzymes, which could be correlated with the Pb-clearance from the cells following oral treatment of the AEEF.

An excess of ROS production promotes apoptotic cell death. Cell apoptosis is initiated by two main principle pathways viz. the mitochondria (intrinsic) mediated and the death receptor (extrinsic) mediated pathways [42]. Mitochondria are both source and target of ROS [43].ROS induced mitochondrial permeability transition, alteration of transcription of signal proteins, and finally DNA fragmentation within the cells. During intrinsic process, pro-apoptotic Bad protein translocates to mitochondria with concomitant down-regulation of antiapoptotic Bcl-2 protein. Later results the discharge of Cyt C into cytosol. Cytosolic Cyt C triggers the cleavage of caspases (active forms) and induces apoptosis [24, 44]. The probable role of ROS in death receptor activation in apoptosis induction has been reported in different literatures [42,24,36]. The Fas system is one of the principle incidences in extrinsic pathway of apoptosis [42]. FAS activation cause immediate recruitment of caspase 8 system resulting cleavage of Pro-caspase 8 into its cleaved fraction. The up-regulation of cleaved caspase 8 enhances the expression of pro-apoptotic Bid [6]. Bid enhances permeability of outer mitochondrial membrane and promotes Cyt C release. On other hand, Cleaved caspase 8 directly activates caspase 3 system [42]. By these ways, extrinsic signals enter into the loop of intrinsic signaling of apoptosis. In current study, immunoblotting revealed the induction of apoptosis following Pb-acetate intoxication. Pb-mediated over-production of ROS may principally instigate the apoptotic cell death. The shielding effect of AEEF may be due to suppression of intracellular ROS production and/or neutralization.

Blood parameters are the formerlydiagnostic indications of pathological states within the body. In our study, elevated quantities of membrane bound enzymes in serum exposed the cellular impairment during Pb-intoxication. The increasedamounts of serum lipids suggested enhanced lipogenesis coupled with reduced excretion of lipoproteins during Pb-intoxication [3,6].The decreased amounts of erythrocytes and haemoglobin can be due to the binding of Pb with erythrocytes [45]. The treatment of AEEF significantly attenuated the Pb-mediated abnormalities of blood parameters which supported the protective effect of AEEF against Pb induced pathogenesis.

In the previous study, the effect of AEEF alone *in vitro* on isolated hepatocytes has been evaluated. However, we did not observe any significant change in the selected parameters with respect to normal control [10]. In sub-acute toxicity study on experimental mice, no significant change was observed in the haematological redox status (S1 and S2 Tables) as compared with normal control group.

Pb-poisoning is known since the history of human cultivation [1]. Industrial revolution coupled with the over-exploitation of Pb containing consumables caused a serious threat of Pb-contamination over past few decades, which finally resulted to a wide range of physiological and behavioral changes to the Pb exposed animals and humans [2]. Pb threat is increasing alarming around the earth including developed nations. Considering the mechanistic aspect of Pb-toxicity, metal chelators + antioxidants would be worthy to protect against Pb-poisoning. However, side effects of commercially available metal chelators largely restricted their clinical applications to human being with variable physiological status [6]. On other hand, a chemically complex edible composition containing both metal chelating agents and antioxidant molecules would, therefore, be beneficial to counteract with Pb-toxicity. Another important aspect of exploitation of edible composition is to achieve the benefit without demonstrable toxic manifestation and/or having minimal effect on normal physiological and biochemical balance (as observed in the effect of AEEF alone). In this study, we explored the beneficial effect of an edible plant, *E*. *fluctuans*, against experimentally induced Pb-toxicity.

## Conclusion

**Fig 14:**
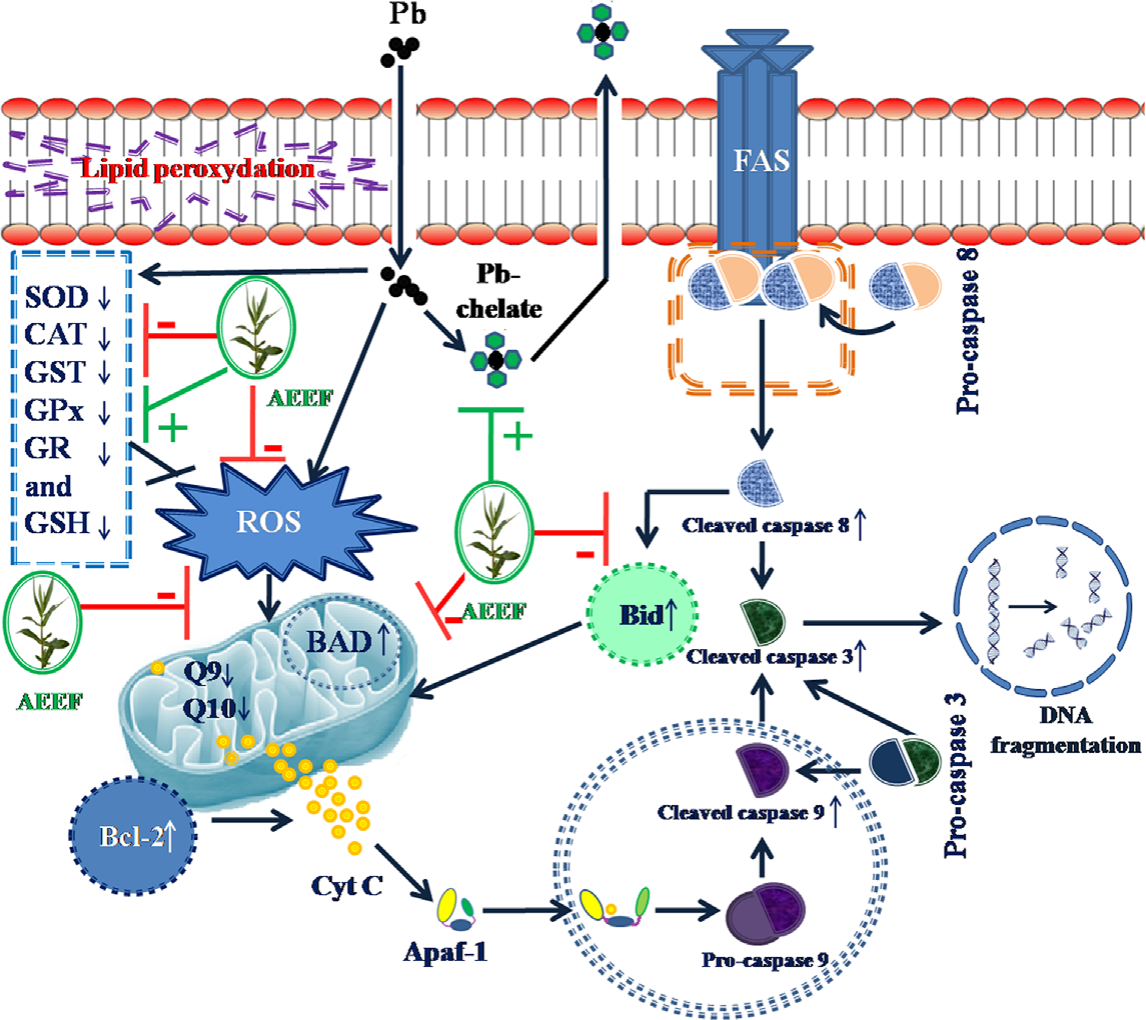
Schematic presentation of the hypothesis developed in this study regarding the overall protective mechanism of AEEF against Pb toxicity. The dark blue arrows indicate the cellular events involved in Pb-induced pathophysiologies. The green lines denoted the activity promoted (+) by AEEF, while, red lines denoted the activity restricted (-) by AEEF.

The Pb toxicity is principally associated with the Pb accumulation within the tissues resulting generation of excessive ROS and inhibition of endogenous oxidative defence system as described previously [6]. Therefore, a combinatorial therapeutic strategy employing Pb chelating agent and antioxidants would be proven fruitful against Pb-intoxication. It would be difficult rather impossible to achieve both this effects from a single molecule. On other hand, multi-component herbal extract could offer multiple health benefits through multi-modal therapeutic offer. In this study, the edible extract of AEEF would offer significant protection against Pb-acetate intoxication in both isolated (*in vitro*) and intact (*in vivo*) systems. AEEF treatment significantly reduced the Pb-content within the selected tissues, which could be correlated with the promotion of Pb clearance following AEEF treatment. Phytochemical analysis indicated presence of phenolics, flavonoids, saponins and ascorbic acid within AEEF. The metal chelating properties of aforementioned phytochemicals have been mentioned in earlier literatures [6,7,46]. However, it was found that still Pb concentration within the tissues remained significantly higher as compared with normal control group. Therefore, Pb-mediated oxidative stress is still obvious. In this study, AEEF could significantly attenuate the Pb mediated ROS generation and counteract with the endogenous redox challenge. Phenolics, flavonoids and ascorbic acid are reputed dietary antioxidants [3,10,36,47] which could significantly contribute in the process of attenuation during Pb-mediated oxidative challenge. Therefore, it could be hypothized that both the metal chelating and antioxidant effect simulteniously contributed in overall protection in a synergy (Fig 14).

## Supporting Information

S1 Table Effect of AEEF (100 mg/kg, p.o.) on haematological parameters of experimental mice. (DOCX)

S2 Table Effect of AEIA (100 mg/kg, p.o.) on ROS production, lipid peroxidation, protein carbonylation, antioxidant enzymes and GSH levels in liver, kidney, heart, brain and testes of experimental mice. (DOCX)
