# Supplementary material for: Cytoprotective and Antioxidant Effects of an Edible Herb, Enhydra fluctuans Lour. (Asteraceae), against Experimentally Induced Lead Acetate Intoxication
Source: PLoS One. 2016 Feb 9;11(2):e0148757. doi: 10.1371/journal.pone.0148757 (PMC4747604; doi:10.1371/journal.pone.0148757)
Supplement: S1 Table — (DOCX) [file pone.0148757.s001.docx]

**Sub-acute toxicity study**

The sub-acute toxicity has been carried out as per established protocol [1, 2]. Two groups of Swiss albino mice (♂, 25 ± 5 g) were selected viz. normal control and the mice were treated with AEEF (100 mg/kg, p.o.) daily for 30 days. On day 31, the animals were sacrificed. Blood samples were collected from retro-orbital venous complex before sacrificing the animals. The organs were removed and rinsed with PBS (pH 7.4) and finally were homogenized in 0.1 M Tris-HCl-0.001 M EDTA buffer (pH 7.4) at 12,000 g for half an hour. The supernatants were used for the analysis of biochemical parameters.

**S1 Table. Effect of AEEF (100 mg/kg, p.o.) on haematological parameters of experimental mice. Normal control data has been used in another manuscript [3].**

| **Groups** | **Haematological and serum biochemical parameters** | **Values** |
| --- | --- | --- |
| Normal control | Total erythrocyte count (x10^6/^mm^3^) | 5.5 ± 0.4 |
| AEEF |  | 5.7 ± 0.6 |
| Normal control | Haemoglobin (g/dl) | 8.9 ± 0.7 |
| AEEF |  | 9.6 ± 0.8 |
| Normal control | Total leucocyte count (x10^3/^mm^3^) | 6.0 ± 0.6 |
| AEEF |  | 5.5 ± 0.7 |
| Normal control | Lactate dehydrogenase (U/l) | 29.2 ± 2.0 |
| AEEF |  | 26.1 ± 1.9 |
| Normal control | Creatinine kinase (IU/ mg protein) | 187.1 ± 11.5 |
| AEEF |  | 178.7 ± 13.5 |
| Normal control | Cholesterol (mg/dl) | 181.5 ± 12.0 |
| AEEF |  | 169.8 ± 11.7 |
| Normal control | Triglycerides (mg/dl) | 112.3 ± 4.2 |
| AEEF |  | 1108.7 ± 7.1 |

Values are expressed as mean ± SE, for ten animals in each group. No significant difference was observed between two groups.

**References**

[1] Witthawaskul, P., Panthong, A., Kanjanapothi, D., Taesothikul, T., Lertprasertsuke, N., 2003. Acute and subacute toxicities of the saponin mixture isolated from *Schefflera leucantha* Viguier. J. Ethnopharmacol. 89, 115-121.

[2] Wills P.J., Asha, V.V., 2012. Acute and subacute toxicity studies of *Lygodium flexuosum* extracts in rats. Asian Pac. J. Trop. Biomed. 2, S200-S202.

[3] Dewanjee S, Dua TK, Khanra R, Das S, Barma S, Joardar S, et al. Water spinach, *Ipomoea aquatica* (Convolvulaceae), ameliorates lead toxicity by inhibiting oxidative stress and apoptosis. Plos One. 201510(10): e0139831. doi:10.1371/journal. pone.0139831.
