# Supplementary material for: Cytoprotective and Antioxidant Effects of an Edible Herb, Enhydra fluctuans Lour. (Asteraceae), against Experimentally Induced Lead Acetate Intoxication
Source: PLoS One. 2016 Feb 9;11(2):e0148757. doi: 10.1371/journal.pone.0148757 (PMC4747604; doi:10.1371/journal.pone.0148757)
Supplement: S2 Table — (DOCX) [file pone.0148757.s002.docx]

**S2 Table. Effect of AEIA (100 mg/kg, p.o.) on ROS production, lipid peroxidation, protein carbonylation, antioxidant enzymes and GSH levels in liver, kidney, heart, brain and testes of experimental mice. Normal control data has been used in another manuscript [3].**

| Parameters | Groups | Liver | Kidney | Heart | Brain | Testes |
| --- | --- | --- | --- | --- | --- | --- |
| ROS production  (nmol DCF/min/ mg of protein) | Normal | 20.3 ± 1.6 | 25.3 ± 1.2 | 20.7 ± 1.9 | 22.5 ± 2.1 | 21.3 ± 1.1 |
|  | AEEF | 19.5 ± 1.0 | 25.2 ± 1.7 | 21.1 ± 1.3 | 21.9 ± 1.9 | 21.8 ± 1.2 |
| Lipid peroxidation  (TBARS level in μg/g of tissue) | Normal | 5.1 ± 0.5 | 5.9 ± 0.3 | 5.7 ± 0.6 | 3.7 ± 0.2 | 4.1 ± 0.2 |
|  | AEEF | 5.1 ± 0.6 | 5.5 ± 0.2 | 5.8 ± 0.5 | 3.3 ± 0.4 | 4.2 ± 0.3 |
| Protein cabonylation  (nmol/mg of protein) | Normal | 32.3 ± 0.9 | 22.4 ± 1.0 | 12.7 ± 0.9 | 9.3 ± 0.7 | 9.0 ± 0.4 |
|  | AEEF | 33.1 ± 1.7 | 22.1 ± 1.3 | 11.9 ± 0.9 | 9.2 ± 0.6 | 9.1 ± 0.7 |
| CAT  (U/mg of protein) | Normal | 208.7 ± 19.7 | 219.0 ± 14.4 | 302.2 ± 20.3 | 104.1 ± 7.6 | 44.1 ± 3.3 |
|  | AEEF | 211.5 ± 14.4 | 224.2 ± 12.7 | 301.1 ± 17.2 | 101.4 ± 6.7 | 43.5 ± 2.7 |
| SOD  (U/mg of protein) | Normal | 117.3 ± 4.9 | 82.1 ± 4.2 | 104.1 ± 5.7 | 80.5 ± 3.3 | 89.7 ± 4.1 |
|  | AEEF | 115.1 ± 6.2 | 79.3 ± 5.5 | 106.5 ± 5.2 | 81.2 ± 6.9 | 88.4 ± 4.5 |
| GPx  (nmol/min/mg of protein) | Normal | 101.6 ± 5.8 | 65.4 ± 2.9 | 178.1 ± 16.0 | 145.1 ± 9.2 | 141.2 ± 7.2 |
|  | AEEF | 98.5 ± 7.1 | 66.2 ± 4.6 | 179.2 ± 12.7 | 144.7 ± 7.2 | 140.1 ± 9.9 |
| GR  (nmol/min/mg of protein) | Normal | 75.1 ± 4.1 | 59.4 ± 3.1 | 72.1 ± 3.9 | 49.2 ± 2.9 | 77.0 ± 3.8 |
|  | AEEF | 73.2 ± 4.7 | 57.4 ± 3.6 | 70.2 ± 4.1 | 47.6 ± 3.3 | 75.7 ± 4.2 |
| GST  (µmol/min/mg of protein) | Normal | 1.1 ± 0.2 | 1.2 ± 0.1 | 0.9 ± 0.05 | 1.2 ± 0.1 | 1.7 ± 0.2 |
|  | AEEF | 1.0 ± 0.05 | 1.1 ± 0.05 | 0.9 ± 0.07 | 1.3 ± 0.05 | 1.8 ± 0.09 |
| GSH  (nmol/mg protein) | Normal  AEEF | 26.9 ± 1.4  27.2 ± 0.9 | 24.0 ± 0.8  25.1 ± 1.5 | 24.8 ± 1.1  25.5 ± 1.2 | 20.1 ± 1.0  20.8 ± 1.2 | 20.6 ± 1.5  20.9 ± 1.6 |

Values are expressed as mean ± SE, for ten animals in each group. No significant difference was observed between two groups.
